# Defining species specific genome differences in malaria parasites

**DOI:** 10.1186/1471-2164-11-128

**Published:** 2010-02-23

**Authors:** Kingsley JL Liew, Guangan Hu, Zbynek Bozdech, Preiser R Peter

**Affiliations:** 1 Division of Genomics and Genetics, School of Biological Sciences, Nanyang Technological University, 60 Nanyang Drive, Singapore 637551, Singapore

## Abstract

**Background:**

In recent years a number of genome sequences for different *plasmodium *species have become available. This has allowed the identification of numerous conserved genes across the different species and has significantly enhanced our understanding of parasite biology. In contrast little is known about species specific differences between the different genomes partly due to the lower sequence coverage and therefore relatively poor annotation of some of the draft genomes particularly the rodent malarias parasite species.

**Results:**

To improve the current annotation and gene identification status of the draft genomes of *P. berghei*, *P. chabaudi *and *P. yoelii*, we performed genome-wide comparisons between these three species. Through analyses via comparative genome hybridizations using a newly designed pan-rodent array as well as in depth bioinformatics analysis, we were able to improve on the coverage of the draft rodent parasite genomes by detecting orthologous genes between these related rodent parasite species. More than 1,000 orthologs for *P. yoelii *were now newly associated with a *P. falciparum *gene. In addition to extending the current core gene set for all plasmodium species this analysis also for the first time identifies a relatively small number of genes that are unique to the primate malaria parasites while a larger gene set is uniquely conserved amongst the rodent malaria parasites.

**Conclusions:**

These findings allow a more thorough investigation of the genes that are important for host specificity in malaria.

## Background

Malaria is a disease caused by the parasitic protozoa from the genus *Plasmodium*. While the disease is restricted to the tropical and sub-tropical regions of the world due mainly to the natural habitat of the mosquito vector, these regions are densely populated with almost 2.2 billion people living in endemic areas and 515 million cases were expected per annum [1]. Although well-established culture and molecular techniques have been established for *Plasmodium falciparum*, the use of rodent malaria parasites as *in vivo *models in the study of the host-parasite interactions is still as relevant today because the rodent parasites are very similar to the human and primate parasites in terms of life cycle, physiology and structure [2].

Since the release of the genome sequences of *P. falciparum *and the rodent malaria species, common features of these haploid genomes include a genome size of 22-26 Mb that are arranged in 14 chromosomes ranging from 0.5-3.0 Mb. In addition the current genomic data show a high degree of conservation between different *Plasmodium *species with the exception of genes located in the telomeric and subtelomeric regions that are extremely variable due to their role in antigenic variation and immune evasion [3,4]. This suggests that genes located close to the centromeres of the chromosomes would be highly conserved. As a proof of concept, comparisons of these centrally located genes between *P. falciparum *and the most completely annotated rodent parasite species *P. yoelii *was shown to have a high degree of synteny [5]. Thus, conservation of these common 'core' genes even amongst divergent species was demonstrated and differences were mainly due to chromosomal re-arrangements [3,4].

In contrast, the chromosomal regions responsible for antigenic variation and host immune evasion show the most divergence. The genome of *P. falciparum *contains species-specific subtelomeric genes involved in host cell invasion, adhesion and antigenic variation that are not found in the *P. yoelii *genome. For example, the *P. falciparum *genes that are located in the sub-telomeric regions include the *var, stevors *and *rifins *that are responsible for antigenic variation and hence immune evasion [6,7]. In contrast, the *P. yoelii yir *gene family seems to be the largest multigene family found in the sub-telomeric regions and is absent in *P. falciparum*. Interestingly, this multigene family is common to all the rodent species and to *P. vivax *[8]. A more recent study employing probabilistic modelling in conjunction with genomic organization and protein structure analysis bridges this gap and places the *P. falciparum rjf/stevor *multigene family together into a conserved multigene superfamily of malaria parasites known as the Plasmodium interspersed repeats (PIRs) [9]. This discovery further suggests that although genes are more conserved than previously thought; there is evidence of species-specific divergence that is dependent on each species' interaction with the host or host immune system. A recent phylogenetic survey of rodent malaria parasites based on DNA sequences from multiple loci in the nuclear, mitochondrial and plastid genomes placed *P. berghei *and *P. yoelii *as sister species forming a distinct clade while *P. chabaudi *and another rodent parasite *P. vinckei *forming another group [10] thus suggesting that *P. berghei *and *P. yoelii *seemed to be more evolutionarily related to each other while *P. chabaudi *appeared to be a more distinct line. While these studies address gene conservation among different species as well as confirm species-specific sequence polymorphisms a global quantitative comparisons with regards to gain or loss of gene function have not been attempted due to the incomplete genome sequences of the malaria parasite species, barring the high resolution map of *P. falciparum *[11]. As a consequence, differences between the rodent parasites especially at the genomic level have not been fully elucidated as yet and thus linking genomic differences to phenotypic traits of these parasites have been difficult. In order to address this issue, genome-wide comparisons including comparative genomic hybridization (CGH) and detailed bioinformatics analysis can be employed as a valuable tool to identify similarities and differences between related species. This technique has been utilized to compare genomic differences between similar species in both prokaryotes and eukaryotes. For example, CGH has been used to genotype related yeast species to determine the presence and absence/polymorphic sequences [12]. The usefulness of CGH in gene discovery was shown with the discovery of three thousand novel genes in *Klebsiella pneuomoniae *342 using microarray hybridization with *Escherichia coli *K-12 open reading frames with confirmation of the presence of genes coding for conserved metabolic functions and demonstrating specificity where genes obtained via lateral transfer in K-12 were absent in 342 [13]. In addition, CGH is also able to differentiate genome-wide variation in various virulent and avirulent *Burkholderia *species [14]. Besides analyzing sequence variation, the gain/loss of DNA can also be used as a tool for elucidating evolutionary divergence between species in their natural environment.

The means for such analysis lies in the use of DNA microarray technology based on the 'spotting' of long oligonucleotides onto glass slides [15-17]. Although the techniques employed for performing DNA microarray experiments have been well established, improvements in the algorithms used for designing the array probes have been critical for advancing the reproducibility and accuracy of detecting the respective gene targets. Recently, a novel robust program called 'OligoRankPick' was able to optimize oligonucleotide design by optimizing target specificity and GC% variation. This algorithm is based on a weighted rank-sum strategy to optimize oligonucleotide selection even along genomes of many diverse organisms [18]. The resultant *P. falciparum *dataset generated from this strategy was shown to be highly reproducible and also led to increased coverage of the *P. falciparum *genome as compared to earlier *P. falciparum *microarray designs. Although the incompleteness of these rodent parasite genomes could present difficulties in their comparative analysis, there is some evidence that they are highly conserved [5]. We have thus emulated the 'OligoRankPick' strategy to design a pan-rodent cross-genome oligonucleotide microarray for the rodent malaria species *P. berghei*, *P. chabaudi *and *P. yoelii*. Due to the incompleteness of the genome sequence available for all three rodent genomes the microarray design approach resulted in oligonucleotide sequences predicted to be complementary to all three rodent species, two rodent malaria species or to be unique to a single species. Comparative genomic hybridization using genomic DNA from each of the three species not only validated the oligonucleotides but at the same time provided new information that allowed the closure of sequence gaps in the genomes of one or more of the rodent species based on complementary hybridization data, thereby significantly improving our overall understanding of gene content for each species. While the CGH approach proofed exceptionally powerful it was still likely that due to sequence polymorphisms found in the different species at the oligonucleotide probe region genes that are actually present would be missed using this strategy alone. For this reason the current available genome sequences were reanalyzed using bioinformatics tools to detect any additional genes missed by the microarray approach alone. Finally, a random selection of genes predicted from the microarray and bioinformatics data to now be present in all rodent genome species was validated using PCR and sequencing.

Overall this multipronged approach allowed us to significantly improve the gene predictions for all three rodent malaria species. In addition the new information obtained in this study allows the assembly of an improved core set of genes present in all plasmodium species while at the same time also identifying a rodent malaria specific gene set.

## Results

### Key design features of the 'Pan-rodent' array chip

**Figure 1:**
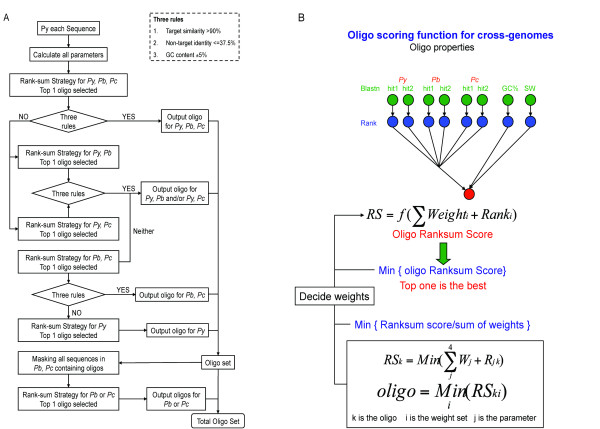
**The overall design schematics of the pan-rodent chip**. (A) Methodology of the chip design. Firstly, all possible oligonucleotides of *P. yoelii *were used to search in the homologous region of the other two species using NCBI blastn and were scored and ranked accordingly. The oligonucleotides were then filtered using three rules such they must have: (i) at least 90% homology to target sequences, (ii) less than 37.5% to non-target sequences and (iii) GC% tolerance of ± 5%. Oligonucleotides for all three species were selected followed by oligonucleotides for *P. yoelii *and *P. berghei *and then for *P. yoelii *and *P. chabaudi*. Next, the remaining oligonucleotides were selected to be specific for both *P. berghei *and *P. chabaudi*. The remaining sequences unaccounted for were then used to design oligonucleotides specific either to *P. yoelii, P. berghei *or *P. chabaudi*. (B) Rank-sum strategy. Oligonucleotides were scored accordingly to (i) first and second BLAST hits, (ii) GC content (Tm) and (iii) Smith-Waterman score (self-binding). The oligonucleotides are then ranked based on each parameter and ordinal rank number is given to all oligonucleotides in each parameter rank independently. The final weighted rank-sum (RS) is calculated for all oligonucleotides using multiple weight sets (not indicated) and the lowest value is considered. Finally, the optimal candidate is selected based on the lowest RS(k) amongst all oligonucleotides in the locus of interest [18]

**Figure 2:**
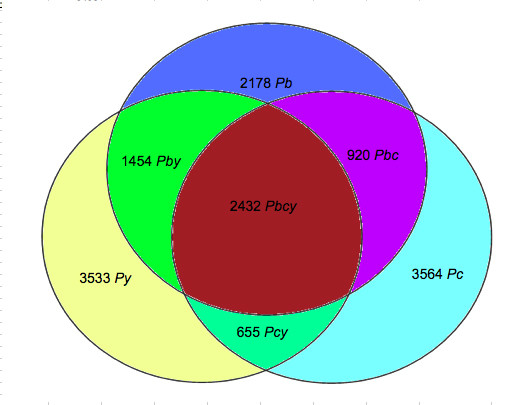
**Venn diagram showing distribution of target rodent parasite gene hits of all oligonucleotides**. All oligonucleotides are 60 bases long and the GC content is targeted at 30% and the allowable deviation is 5% for overlapping oligonucleotides. Complementary oligonucleotides to each rodent malaria parasite species was calculated from the sum of all possible combinations, i.e. oligonucleotides specific to itself and those that can hybridize to itself and to other rodent malaria parasite species. (Legend: *Pb *= *P. berghei *specific oligonucleotides only; *Pc *= *P. chabaudi *specific olinucleotides only; *Py *= *P. yoelii *specific oligonucleotides only; *Pbc *= *P. berghei *&*P. chabaudi *specific oligonucleotides; *Pby *= *P. berghei *&*P. yoelii *specific oligonucleotides; *Pcy *= *P. chabaudi *&*P. yoelii *specific oligonucleotides; *Pbcy *= oligonucleotides specific to all 3 rodent parasite species)

In order to detect genes from all three rodent malaria parasite (RMP) species, the DNA microarray design was based on the 'OligoRankPick' program [18] as this algorithm was shown to have significant improvements over other strategies even when dealing with large fluctuations in GC content and abundant gene duplications. Since the microarray designed here was to include all available information for three RMP species, there are key challenges and modifications to our original program so as to accommodate multiple genomes on a single microarray. Since *P. yoelii *is the only RMP with a published draft genome [3], we first designed oligonucleotides against the predicted *P. yoelii *gene models. Next, all possible oligonucleotides of *P. yoelii *were used to query the homologous regions of the other two RMP species (Figure 1A). Next, four parameters were used to select for the best oligonucleotide, namely: BLAST scores (first and second hit), GC content, sequence complexity and self-annealing scores (Figure 1B). Each score is then transformed into a rank and a weighted rank-sum is calculated for each oligonucleotide with the final oligonucleotide being selected based on the smallest rank-sum value. These oligonucleotides were then selected to be optimal for all three species, followed by oligonucleotides for *P. yoelii *and *P. berghei *and then for *P. yoelii *and *P. chabaudi *(Figure 1A). For the remaining predicted open reading frames oligonucleotides specific for each species were then designed. A total of 14,736 oligonucleotides were thus obtained and the breakdown is shown in Figure 2.

### Comparative Genomic Hybridization using the Pan-rodent microarray

Since some of the oligonucleotides are designed to be capable of cross hybridizing with more than one species, we have re-sorted all the genes from the three species and matched them with their respective oligonucleotides so that an accurate normalized value can be obtained for analysis. Using this approach, we performed comparative genomic hybridization experiments and first looked at the performance of the species-specific oligonucleotides that were designed to hybridize with their target genome. After normalizing and filtering the data the percentage of successful oligonucleotides for *P. yoelii-*specific, *P. berghei*-specific and *P. chabaudi*-specific oligonucleotides were 91% (7,347), 90% (6,314) and 84% (6,356) respectively. It is not surprising that a low proportion of oligonucleotides were unable to hybridize with their intended targets especially if they were designed in regions with low confidence in sequence quality. A likely reason is the relatively poor genome coverage of the rodent parasite genomes as compared to the more complete *P. falciparum *genome. Also, there are errors in the current sequence drafts of the rodent malaria parasites due to the propensity of random sequence rearrangements of an AT-rich genome in a sequencing vector.

Using the validated set of oligonucleotides, we identified all the oligonucleotides that hybridized to the DNA of parasites they had not originally been designed against (Figure 2). A positive hybridization signal provides strong evidence that the sequences and therefore the genes represented by these oligonucleotides are also present in the genomes of the other rodent parasite species. This strongly implies that the respective genes that are represented by these oligonucleotides are actually present but are not found in the current database.

### Filling the gaps in the genomes of the rodent malaria parasite species

Since it has been established that the malaria parasite genomes are well conserved [5], it is conceivable that genes that are missing in the current draft of either of the RMP genome have a well conserved ortholog in one or two of the other species. Using the pan-rodent microarray we wish to investigate this possibility by inspecting comparative genome hybridization (CGH) signals on "cross-species" oligonucleotide microarray elements. For example high signal from *P. yoelii *gDNA for an oligonucleotide that is not designed to hybridize to *P. yoelii *implies that this gene (sequence) is present in *P. yoelii*. In summary, a species where the draft genome currently does not possess a particular gene but now gives a signal on the array suggests the presence of this orthologous gene. Hence, this gene can thus be detected based on homology.

Missing genes in the draft genome could arise due to two scenarios: either the sequence information is missing, or that the sequence is present in the genome but missed by current gene prediction algorithms. Since the oligonucleotides were designed based on predicted open reading frames, a CGH signal constitutes direct experimental evidence for the presence of an orthologous genomic sequence and thus potentially the gene in the RMP genome in which this gene is missing. Based on this approach, we detect 179, 306 and 215 genes missing in the current draft sequences of the *P. yoelii*, the *P. berghei *and the *P. chabaudi *genome, respectively. The majority of these genes code for hypothetical proteins that lack any functional assignment based on their amino acid sequence. For those whose function has been described, genes involved in biosynthesis, protein modifications, kinases and also invasion-related proteins in the case of *P. chabaudi *and *P. berghei *have been discovered (Additional file 1: Supplemental Table S1).

**Figure 3:**
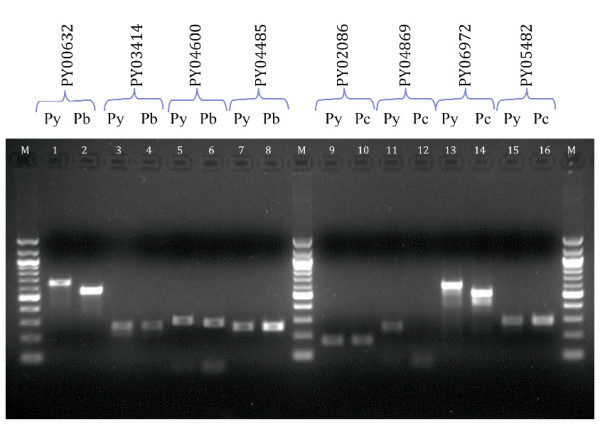
**PCR screening of a random sample of newly discovered genes**. Screenings were performed pair-wise with the PCR products of the species containing the known gene of interest loaded in odd-numbered wells while the corresponding PCR screen of the other species whereby sequence is absent or the gene is not predicted are in the even-numbered wells. The Genbank accession numbers of these novel orthologs are indicated in parentheses in the following description. (1&2): PY00632 screen with Py and Pb gDNA (GU390534); (3&4): PY03414 screen with Py and Pb gDNA (GU390535); (5&6): PY04600 screen with Py and Pb gDNA (GU390540); (7&8): PY04485 screen with Py and Pb gDNA (GU390538); (9&10): PY02086 screen with Py and Pc gDNA (GU390539); (11&12): PY04869 with Py and Pc gDNA; (13&14): PY06972 screen with Py and Pc gDNA (GU390536); (15&16): PY05482 screen with Py and Pc gDNA (GU390537). (Legend: M = 100 bp DNA ladder)

A selection of genes that were detected via the microarray data were randomly selected for polymerase chain reaction (PCR) screening (Figure 3) and direct sequencing in order to establish the confidence of this group of newly discovered genes. PCR primer pairs were designed flanking the oligonucleotide sequence to the species where there is known sequence information and these were used to amplify a newly predicted gene in another species where it is not annotated/predicted or where the sequence is absent. All screens were performed on regions where sequence information for the newly discovered orthologous genes is not present (i.e. missing sequence) except for PY00632 and PY03414 where a corresponding *P. berghei *contig is present but the gene is not predicted. In summary, 7 of the 8 PCR reactions gave a product around the predicted size and the one sample that was PCR negative could be due to sequence polymorphisms at the primer sites. Sequence analysis of the PCR product confirmed that the PCR product did indeed represent the predicted gene (data not shown). Some PCR products also exhibit a change in the predicted size, for example different PCR product sizes of PY00632 and its *P. berghei *ortholog are expected based on the currently available sequence information. Differences in PCR product size are due to variations in sequence length in the region bounded by the primers. The differences in PCR product size in the PY06972 screen of *P. yoelii *and *P. chabaudi *genomes are also due to the same reason. The high congruence of PCR-positive screens showed the power of the array in detecting homologous sequences currently absent from the available genome sequences of the other species. Additional microarray investigations pertaining to the detection and validation of polymorphic genes and differential transcription profiles between related parasite clones have further validated the performance of the oligonucleotide probes (unpublished).

### Cross-species gene identification using bioinformatics

**Figure 4:**
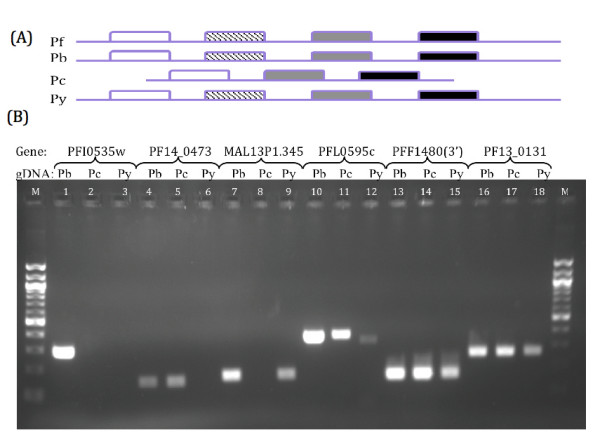
**PCR screening of bioinformatics-filtered dataset with contig information**. (A) Schematic depicting the scenario whereby a contig from 1 species containing the gene of interest (shown as a hatched block) is aligned with the best corresponding hit contig from another species. In this case, the gene of interest is lost in 1 species while the flanking sequences are still present. (B) PFI0535w-PB104921.00.0 orthologs screened with (1)Pb, (2)Pc and (3)Py gDNA. PF14 0473-PC0001359.02.0 orthologs screened with (4)Pb, (5)Pc and (6)Py gDNA. MAL13P1.345-PY04218 orthologs genes screened with (7)Pb, (8)Pc and (9)Py gDNA. PFL0595c-PB301230.00.0-PC000699.01.0 orthologs screened with (10)Pb, (11)Pc and (12)Py gDNA. PFF1480w(3'end)-PB000730.00.0PY03519 orthologs screened with (13)Pb, (14)Pc and (15)Py gDNA. PF13_0131PC000708.04.0-PY04599 orthologs screened with (16)Pb, (17)Pc and (18)Py gDNA. (Legend: M = 100 bp DNA ladder)

**Figure 5:**
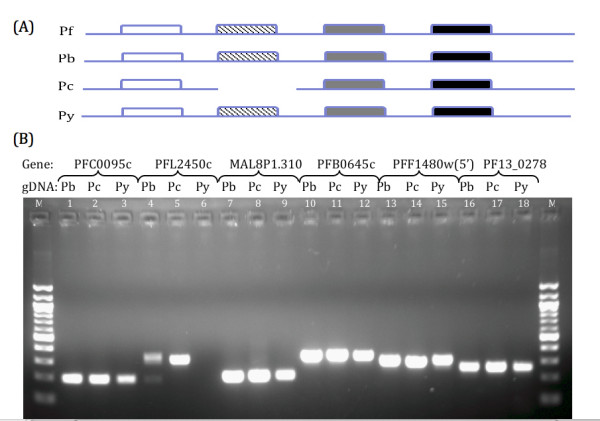
**PCR screening of bioinformatics-filtered dataset without contig information**. (A) Schematic depicting the scenario whereby a contig from 1 species containing the gene of interest (shown as a hatched block) is aligned with the best corresponding hit contig from another species. In this case, the gene of interest is lost in 1 species due to missing sequence information while the adjacent flanking contig sequences are still present. (B) PFC0095c-PB000276.02.0 orthologs screened with (1)Pb, (2)Pc and (3)Py gDNA. PFL2450c-PC000344.03.0 orthologs screened with (4)Pb, (5)Pc and (6)Py gDNA. MAL8P1.310-PY06565 orthologs screened with (7)Pb, (8)Pc and (9)Py gDNA. PFB0645c-PB000193.00.0-PC000452.02.0 orthologs screened with (10)Pb, (11)Pc and (12)Py gDNA. PFF1480w(5'-end)-PB000730.00.0-PY03519 orthologs screened with (13)Pb, (14)Pc and (15)Py gDNA. PF13_0278-PC000860.02.0-PY04659 orthologs screened with (16)Pb, (17)Pc and (18)Py gDNA. (Legend: M = 100 bp DNA ladder)

Although a total of 700 novel orthologous rodent parasite genes have been discovered via the array approach, these revisions still leave significant gaps in the genomes of the three rodent parasite species. Therefore in addition to CGH, we also utilize bioinformatics to look at the genome in its entirety so as to probe for open reading frames that have been missed by the automated gene predictions provided by the RMP sequencing projects. The use of bioinformatics tools to query non-coding regions of the rodent malaria parasite genomes can be useful in detecting genes that have been missed by automated gene prediction algorithms. The more complete *P. falciparum *amino acid sequences were used to query the rodent malaria parasite genomes as a tBLASTn search using an expect value of at least 10^-15 ^as a cut-off threshold. While this approach does not account for synteny, evidence that regulation of transcription of individual genes occurred independently without any constraints on chromosomal location further supports this approach [19,20]. In addition, multigene families would be collapsed, as conserved domains would link such members together. Using this approach, 103 orthologous genes were discovered in *P. berghei*, 93 in *P. chabaudi *and 286 in *P. yoelii*. This data also suggests that while the *P. yoelii *genome is more completely assembled, it is lacking a number of predicted open reading frames. Similarly, PCR screens of these predicted genes were performed in order to obtain additional confidence for the bioinformatics derived dataset. For this confirmation, two groups were selected based on whether a homologous contig for a particular species is present in a region where a gene is not predicted, thereby checking if this gene is truly deleted from this locus or is still present, being either translocated to another locus or missed by the automated contig assembly (Figure 4). On another hand, we have observed another scenario where a newly discovered orthologous gene is not present in the species of interest. While sequence information is absent for these candidate genes, the adjacent genes are present in separate contigs. We thus wanted to screen these gaps or assembly errors that have excluded this gene (Figure 5). The re-annotated *P. chabaudi *gene orthologous to PFF1480w, PB000730.00.0 and PY03519 was screened twice as the best-fit *P. chabaudi *contig only contains the 5'-end sequence of this gene while the 3'-end is missing. Based on the random sampling approach (Figure 4), the PFI0535w-PB104921.00.0 pair seems to be exclusively found in *P. berghei *and not in the other 2 RMP species. For the PF14 0473-PC0001359.02.0 pair, it seems that this gene is also present in *P. berghei *while the MAL13P1.345-PY04218 pair looks to be also present in *P. berghei *as well. As for the other three pairs of genes (PFL0595c-PB301230.00.0¬PC000699.01.0; PFF1480w(3'end)-PB000730.00.0-PY03519; PF13_0131¬PC000708.04.0-PY04599) that seem to be missing in either one of the three RMP species, PCR screening suggests otherwise and that they are indeed common to all three RMP species. These results suggest that rodent parasite genes that are seemingly absent on a contig are likely to be present with high confidence in all three rodent malaria parasite species. Almost all of the other panel's screenings (Figure 5) showed that all of those genes were present in the three RMP species barring the *P. chabaudi *exclusive gene (PFL2450c-PC000344.03.0) that seemed to be present also in *P. berghei *but seemingly not in *P. yoelii*. This suggests with high confidence that the missing genes due to incomplete sequence information are present. Overall, it seems that it is highly likely that missing genes due to poor sequencing coverage are present and genes common to two rodent malaria parasite species are very likely to be present in all three species.

### Defining the core *Plasmodium *genome

With the more complete coverage of the rodent malaria parasite genomes obtained via the analysis here, we now thought to define a common 'core' *Plasmodium *genome where orthologous genes present in all sequenced malaria parasites was defined. *Plasmodium falciparum*, being the most completely annotated and researched malaria parasite genome, is used as an index reference species in an attempt to consolidate genes common to as many species as possible. For each *P. falciparum *gene the orthologs present in the rodent parasite species were matched, and the resulting dataset was further appended with the respective orthologs from another human malaria parasite *P. vivax *and the simian parasite *P. knowlesi *as these genomes had been recently sequenced to 10× and 8× [21,22] coverage respectively. This approach segregated *P. falciparum *genes into two groups: firstly, the 'core' genes containing orthologs to the rodent parasite as well as the *P. vivax *and *P. knowlesi *sequences (Additional file 2: Supplemental Table S2) and secondly, genes that have no significant alignment with rodent sequences (Additional file 3: Supplemental Table S3). While orthologous genes (with respect to *P. falciparum*) are highly conserved in regions where housekeeping genes predominate, the telomeric and sub-telomeric regions (i.e. non-syntenic) contain genes involved in antigenic variation that are more divergent [3] and this is consistent with the observation that the group representing the non-orthologous genes is dominated by *var, stevor *and *rifin *gene families that are involved in antigenic variation. What is immediately apparent is the high similarity of all six parasite species in the regions that contain housekeeping genes. In total, 4,188 genes were common between *P. falciparum *and the rodent parasites. Of these, 73 genes, mainly hypothetical genes, were absent in both *P. vivax *and *P. knowlesi*. In addition, 50 genes were present in *P. vivax *but not in *P. knowlesi *and 79 genes were present in *P. knowlesi *but not in *P. vivax*. Without these species-specific genes, we found 3,986 genes that are common to all six species.

An additional 387 *P. falciparum *genes (conserved in the two primate malaria parasites) appear to be missing in one or two of the rodent malaria parasite species (Table 1) with the vast majority of these representing hypothetical genes. The main exception represents genes conserved between *P. falciparum*, *P. berghei *and *P. yoelii *but missing in *P. chabaudi *from which approximately 30% have a functional annotation. Of the 387 genes, only a relatively small number (27-40) are exclusively found in only *P. falciparum *and one of the rodent malaria species with a significantly large number (92-96) being present in two species. While the genes missing in one or two rodent species could potentially represent species specific gene deletions, the fact that a significant number of these (72%-96%) orthologs are also present in the more divergent *P. knowlesi *and *P. vivax *genomes are highly suggestive that the respective rodent orthologs that have not yet been identified by either the sequencing project or the analysis reported here could likely be present.

**Table 1:** Syntenic Pf genes with corresponding RMP chromosome.

Gene groups	No. of genes	Hypothetical genes	Corresponding Pv genes	Corresponding Pk genes
Pf	46	45 (98%)	28 (61%)	30 (65%)
Pf-Pb	40	38 (95%)	31 (78%)	32 (80%)
Pf-Pc	27	25 (93%)	22 (81%)	24 (89%)
Pf-Py	36	34 (94%)	26 (72%)	28 (78%)
Pf-Pb-Py	96	67 (70%)	87 (91%)	90 (94%)
Pf-Pb-Pc	96	77 (80%)	91 (95%)	93 (97%)
Pf-Pc-Py	92	78 (85%)	88 (96%)	88 (96%)

Gene clusters of orthologous genes across six malaria parasite species, namely *P. falciparum *(Pf), *P. berghei *(Pb), *P. chabaudi *(Pc), *P. yoelii *(Py), *P. knowlesi *(Pk) and *P. vivax *(Pv). More genes are common to all six species where evidence of rodent malaria parasite (RMP) chromosome matching to *P. falciparum *syntenic regions is present.

On the other hand we identify 927 *P. falciparum *genes without a match to any rodent parasite species, 469 were composed of hypothetical genes (Table 2), with the majority of known genes being made up of the *var, rif *and *stevor *multigene families that are present at the subtelomeric regions of essentially all *P. falciparum *chromosomes (Additional file 3: supplemental table S3). Only about 14% of the 927 genes were conserved in *P. vivax *and/or *P. knowlesi*, with 117 being present in both species. While all genes exclusive to *P. falciparum *and *P. knowlesi *represent hypothetical genes, 6 genes specific to *P. falciparum *and *P. vivax *encode proteins involved in parasite invasion; 2 cysteine proteases and 4 MSP7-like genes. Taken together we define 4,188 genes that form a core gene set for a Plasmodium genus. From this set only a small fraction of genes (27 to 96 ~ 0.6%-2.2%) is lost in one or two of the six *Plasmodium *species. In contrast there are a large number of genes that appear specific to individual *Plasmodium *species and conserved only in one or two others. These mainly involve gene associated with host parasite interaction and are localized in the subtelomeric regions. However, limited diversification was also observed in genes present in the intrachromosomal region mainly involved in other Plasmodium specific functions such as invasion.

**Table 2:** Non-syntenic Pf genes without corresponding RMP chromosome.

Gene groups	No. of genes	Hypothetical genes	Corresponding Pv genes	Corresponding Pk genes
Pf	927	469 (51%)	129 (14%)	120 (13%)
Pf-Pb	0	0	0	0
Pf-Pc	0	0	0	0
Pf-Py	1	0	0	0
Pf-Pb-Py	1	1 (100%)	0	0
Pf-Pb-Pc	1	1 (100%)	0	0
Pf-Pc-Py	0	0	0	0

Gene clusters of orthologous genes across six malaria parasite species, namely *P. falciparum *(Pf), *P. berghei *(Pb), *P. chabaudi *(Pc), *P. yoelii *(Py), *P. knowlesi *(Pk) and *P. vivax *(Pv). In contrast to genes found in syntenic regions (Table 1) significantly less genes are common in *P. falciparum *species specific coding regions.

### Defining the core rodent *Plasmodium *genome

**Figure 6:**
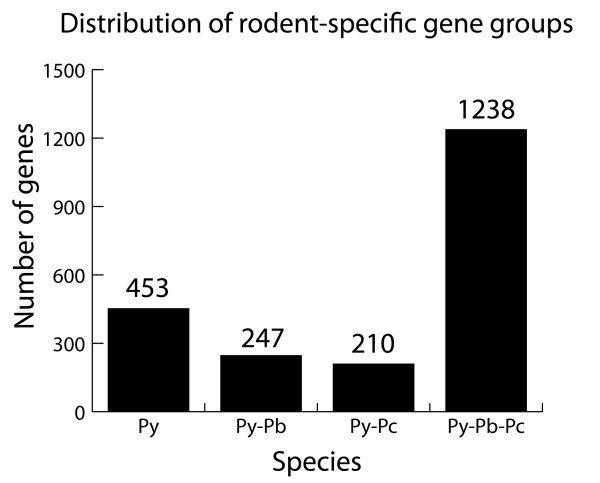
**Graphical plot showing the re-distribution of the rodent malaria parasite genes after removing the PIR genes**. Most of the reduction occurs in the group Py-Pb-Pc in which all the three rodent malaria parasite species share a common gene. (Legend: Py = genes specific to *P. yoelii*; Py-Pb = genes common to both *P. yoelii *and *P. berghei*; Py-Pc = genes common to both *P. yoelii *and *P. chabaudi*; Py-Pb-Pc = genes common to all three species)

Using a similar approach as in the previous section, we attempt to construct a rodent parasite specific associative table since we can also take advantage of the dataset from the genomic hybridizations. For this purpose, instead of using genes from *P. falciparum *as an index, the oligonucleotides were chosen as the index and then replaced with the best-hit *P. yoelii *gene. Next, *P. falciparum *- *P. yoelii*, *P. berghei *- *P. yoelii *and *P. chabaudi *- *P. yoelii *orthologs were appended to the list. This strategy creates a database of genes orthologous to *P. yoelii *and the reason for choosing this species as the index is due to it being more completely annotated than the other two rodent parasite species. This list was refined by removing matching orthologs from *P. falciparum *that were already present in the common 'core' set; thereby creating a filtered list that contains only genes that are specific to the rodent malaria parasites. Microarray data and bioinformatics (tBLASTn) to query the *P. yoelii *amino acid sequences were also employed. In order to reduce the complexity of the dataset, the PIRs, which consist of the largest multigene family and dominate the rodent specific genes, will not be discussed due to their variability of expression and gene expansion amongst the rodent malaria parasites due to its main role in antigenic variation [23,24] as well as its copy number being the most abundant in *P. yoelii*. Consequently, the huge reduction in genes common in all three rodent malaria parasite species is due to the removal of the PIR genes that could also confound relationships based on bioinformatics simply due to multiple matching of common conserved domains (Additional file 4: Supplemental Table S4; Figure 6).

Overall this approach now identified 1,238 genes that are common to all rodent malaria species most of which (1,013) represent hypothetical genes. Importantly only about 8% of these predicted genes have ORF that are shorter than 100 nucleotides making it less likely that these are annotation or prediction errors but indeed represent functional genes. Taking together these gene numbers along with the 4,188 genes found in the 'core' set, the total number of conserved rodent malaria genes is 5,426. In addition it appears that there are 210 genes specific to *P. yoelii *and *P. chabaudi *(Additional file 5: Supplemental Table S5), 247 genes specific to *P. yoelii *and *P. berghei *(Additional file 6: Supplemental Table S6) and 453 unique to *P. yoelii *(Additional file 7: Supplemental Table S7) with most of them (> 90%) again representing hypothetical genes. Of the 453 unique *P. yoelii *genes 45% are less then 100 nucleotide in length, compared to 24% in the *P. yoelii *and *P. berghei *group and 17% in the *P. yoelii *and *P. chabaudi *group indicating that a number of these genes represent false gene models.

## Discussion

Our microarray specific to the rodent malaria species *P. berghei, P. chabaudi *and *P. yoelii *has provided a golden opportunity to verify the genomes of these rodent malaria parasite (RMP) species. The RMP draft genomes were annotated similarly where GlimmerMExon was first trained on *P. falciparum *data and automated assignments were based on hidden Markov model association [3,25]. Since these genomes were annotated in a similar manner, we do not foresee any annotation bias in the three RMP draft genomes. Our oligonucleotide selection process aims to include every predicted gene model and the robustness of the design algorithm has been shown in the ability to differentiate between members of large multigene families [18]. Evidence of significant homology via hybridization and bioinformatics show that a high proportion of these gene models are conserved amongst the three RMP species, therefore suggesting that a high proportion represent true gene models that are refractory to random mutations as compared to intronic sequences.

The definition of the core gene set of *Plasmodia *genes using the most completely annotated genome of *P. falciparum *as a reference index has enabled the use of direct hybridization and bioinformatics tools to expand the repertoire of common genes. The validity of utilizing CGH and bioinformatics to improve genome annotation is well established [12-14,26]. Novel gene models discovered via hybridization have been validated with PCR (Figure 3) while genes discovered via both hybridization and bioinformatics were also validated (Figures 4 and 5). In fact, PCR screens of a subset of genes without at least one RMP ortholog suggest that there is high confidence that any *P. falciparum *gene that contains at least an ortholog from one of the RMP species can potentially contain orthologs from all three RMP species. This suggests that the high stringency thresholds in both hybridization and bioinformatics are in fact conservative and there are potentially more genes that have yet to be discovered. Hence, the results indicate that the genetic repertoires of the RMP species are indeed more common and that these genes should be present in the poorly assembled and annotated rodent parasite genomes.

The construction of a rodent specific orthology map using *P. yoelii *as the reference index was undertaken to study and survey rodent parasite specific genes that are distinct from the common 'core' set. Although this process would filter off genes specific to the other two RMP species, the *P. yoelii *genome was chosen as it is the most completely annotated rodent parasite genome while the gene set in *P. chabaudi *is over-predicted thus making comparisons across species difficult. This associative table clearly demonstrates certain species-specific metabolic differences and reveals gene duplications and expansions from both the core set of *Plasmodia *genes and the rodent parasite specific genes. Similarly, hybridization data reveals more orthologous genes than the current known set.

Due to the high AT-bias in the genome of the *Plasmodia *species, sequencing the entire genome, joining overlapping sequences to form contigs and gene predictions have been difficult. While the majority of the rodent malaria parasite genes have been sequenced and annotated, many gaps still remain and the data from the pan-rodent cross-genome oligonucleotide microarray provides direct experimental evidence for this case. While the genomes from *P. berghei *and *P. chabaudi *continues to be refined at the Sanger Institute, improvements on the sequence coverage of the *P. yoelii *genome has not been taken up by any group. Therefore, any improvements in genome coverage for *P. yoelii *would be extremely valuable.

More importantly, the total number of orthologous gene pairs obtained in this survey (Table 3) is now significantly higher than the published dataset [25] except for the *P. berghei *and *P. chabaudi *orthologous gene pair where they have been over-predicted. The total number of ortholgous pairs is calculated by the sum of the core genes together with the remaining *P. falciparum *- RMP gene permutations as well as any RMP specific genes. For example, for the *P. yoelii *vs. *P. falciparum *orthologous gene pair, the sum is 4,188 (core Pf-RMP) + 37 (Pf-Py) + 97 (Pf-Pb-Py) + 92 (Pf-Pc-Py) = 4,414. PCR screening also strongly suggests that a *P. falciparum *gene with at least a single RMP ortholog is also likely to contain orthologs from all three RMP species. Since these genes total up to 4,578, the potential maximum of orthologous genes will be computed using this assumption where the 4,188 core genes are replaced by the theoretical maximum of 4,578 genes. This work augments and complements the recent manual re-annotation of *P. yoelii *genes [26] and the release of the dataset presented here is intended to provide a reference resource for researchers working on the in vivo rodent malaria species model, especially where predicted orthologs are currently unavailable. Data from both the core and rodent species specific gene datasets can now form the basis for investigating the differences in host cell selectivity and other pathophysiological traits such as sequestration observed between the different RMP. It is not surprising that the majority of the rodent species specific genes have no known function but by now having identified a relatively small number of genes, a more thorough investigation to qualitatively associate specific genes with species specific traits is feasible. On the other hand this study does not exclude that differences in the expression of conserved genes also contributes to species specific differences.

**Table 3:** Orthologs between *P. falciparum *and three rodent malaria parasite species.

	***Pb vs. Py***	***Pb vs. Pc***	***Pc vs. Py***	***Py vs. Pf***	***Pb vs. Pf***	***Pc vs. Pf***
'Core' set	4188	4188	4188	4188	4188	4188
<3 RMP orthologs	-	-	-	226	234	215
RMP specific	1485	-	1448	-	-	-
**Total**	**5673**	**4188**	**5636**	**4414**	**4422**	**4403**
**Current set**	**3153**	**4641**	**3318**	**3375**	**3890**	**3842**
Potential 'Core' set	4578	4578	4578	4578	4578	4578
Potential total	6063	4578	6026	4578	4578	4578

Summary table of orthologs between *P. falciparum *and the three rodent parasite species. The 'core' set represent orthologs common in all four species defined via hybridization and bioinformatics information. Other gene set combinations were taken from the remaining permutations of genes generated from those with at least 2 RMP orthologs as well as the rodent parasite specific genes. The total number of orthologous pairs is the sum of the core genes with the other respective gene permutations while the current set denotes the status quo [25]. The potential total indicates the theoretical maximum of common genes as there is strong suggestion that a *P. falciparum *gene with at least one RMP ortholog can potentially be orthologous to all three RMP species.

Our approach here provides a significant improvement of gene coverage using existing information and well-established experimental and analysis techniques. The additional information provided here will be of particular use for future efforts using next-generation DNA sequencing technologies.

## Conclusions

Unlike most previous studies that have focused on finding similarities between the genomes of different plasmodium species [3,5,21,22,25] the work here for the first time attempts to define the difference in gene content between the different parasites. Currently, our understanding on the genetic basis for species specific restriction of malaria parasites is limited. This is mainly due to the incomplete status of the RMP genomes that do not allow reasonable assessment of their nuclear encoded proteomes. Conducting the cross species CGH and the reciprocal homology searches allowed us to generate substantially improved dataset that enables us for the first time to investigate the subsets of genes that may be involved in *Plasmodium *speciation. First, we identify 117 genes that are found in only the human and primate malaria species should be considered key candidates for separating rodent from primate malarias. Similarly the 1,238 RMP specific genes are likely to provide the biological framework that separates the rodent from primate malaria. How these mainly hypothetical genes function in the parasite biology now needs to be further investigated.

In contrast to the relatively large numbers of genes that are unique to individual species (e.g. 927 gene unique to *P. falciparum*) or species subgoups (e.g. 1,238 specific to RMP), there is only a small number of genes that are lost from the generic core gene set 27 to 96 genes lost in one or two Plasmodium sp.). With the rodent parasites forming a distinct phylogenetic clade and genetic evidence that *P. vivax *and *P. knowlesi *are more similar to each other [27,28] this suggests that species specific gene expansion and diversification is the main driving force of speciation amongst *Plasmodium *species. This is likely to apply to genes responsible for invasion and antigenic variation, but also genes involved in other species specific phenotypes such as the asynchronous character of the intra-erythrocytic development of *P. yoelii*, the formation of hypnozoites in *P. vivax *and the propensity of sequestration.

## Methods

### Microarray fabrication

A modification of the 'OligoRankPick' program [18] was employed to design 60-mer probes to the three rodent parasite species *P. berghei, P. chabaudi *and *P. yoelii *(Additional file 8: Supplemental Table S8; Additional file 9 - Supplementary Table S9) using sequences deposited in PlasmoDB http://www.plasmodb.org. Cross-species oligonucleotides were selected based on the criteria as described in the results section, i.e. at least 90% homology to the target sequence, less that 37.5% to non-target sequences and capped with a 5% difference tolerance in GC content. All possible oligonucleotides for a particular gene was then ranked and sorted based on target (>90%) and non-target hit (<37.5%), GC content (±5%) and the best Smith-Waterman score (Figure 1). Hence, all possible oligonucleotides were evaluated based on every available gene model and the best sequence was then selected. Oligonucleotides were spotted onto poly-L-lysine-coated microscopic glass slides [15].

### Rodent parasite DNA preparation for genomic DNA microarray

Balb/c mice were used as vertebrate hosts for the propagation of *P. berghei *ANKA, *P. chabaudi *AS and *P. yoelii *17 × 1.1. All procedures were in accordance with the guidelines for the use of experimental animals established by the Institutional Animal Care and Use Committee (IACUC) of the Nanyang Technological University of Singapore. Leukocytes were filtered from whole blood [29] and gDNA extracted using the Easy-DNA™ kit (Invitrogen) according to the manufacturer's protocol. For each parasite line, 3 μg of DNA was mixed with 2.5 μg of random nanomer primer to a volume of 15.25 μl. DNA was denatured at 100°C for 5 min and snap cooled on ice for 5 min. Labeled DNA was generated by adding dNTPs to a final concentration of 1 mM dATP and 500 fM each: dCTP, dGTP, dTTP and 5-(3-aminoallyl)¬2'-deoxyuridine-5'-triophosphate, (aa-dUTP) (Biotium), with 20 units of exo-Klenow Fragment (New England Biolabs) in a total volume of 50 μl and incubated at room temperature for 10 min and then overnight at 37°C. Cy-dye coupling, cleanup, hybridization, washing and slide scanning were performed as described by Bozdech and co-workers [15]. Experiments were performed with at least duplicates for each species Subsequently, the data were normalized using the NOMAD microarray database http://ucsf-nomad.sourceforge.net/. For the comparative genome analysis, low quality features and features with a signal level less than two-fold of the background plus two-fold of the background standard deviation was filtered from the initial raw data set.

### Polymerase Chain Reaction (PCR)

Verification of genes was performed via PCR thermocycling (Eppendorf) using the following conditions: 1 cycle of 95°C for 2 min; followed by 35 cycles of 95°C for 1 min, 50°C for 1 min and 68°C for 30 s. A final extension was set for 10 min at 68°C and then the tubes were kept at 4°C. For each 20 μl reaction, 2 ng of DNA was used as a template with 10 pmol of the forward and reverse primers (Additional file 10: Supplemental Table S10), 200 μM of dNTPs with 1.5 units of Taq DNA polymerase (Kapa Biosystems).

### Bioinformatics tools

The sequences of *P. falciparum, P. knowlesi *and *P. vivax *were obtained from PlasmoDB http://www.plasmodb.org. For any gene missing an orthologous partner in another species even after complementation with the array data, known orthologs were appended using the resource available at PlasmoDB (version 5.5). Unannotated genes were identified using the tBLASTn search where amino acid sequences from a well-annotated species (PlasmoDB version 5.5) are used to query the genome of another species. A threshold of 10^-15 ^was used to ensure the stringency of locating matching genes.

### Availability of microarray data

Microarray data are publically available at the Centre for Information Biology Gene Expression Database (CIBEX; http://cibex.nig.ac.jp). The Accession number for these data is CBX114.

## Authors' contributions

KL designed and carried out the experiments and analyzed the data and helped with the writing of the manuscript. GH designed the microarray and helped with the analysis of the array data. ZB helped with the design of this study and helped in the writing of the manuscript. PRP designed and supervised the study and wrote the manuscript. All authors read and approved the final manuscript.

## Supplementary Material

Additional file 1 **Supplemental Table S1**. Identification of orthologs by microarray. Click here for file

Additional file 2 **Supplemental Table S2**. Orthologous genes in *P. falciparum *and RMP. Click here for file

Additional file 3 **Supplemental Table S3**. *P. falciparum *genes with no RMP ortholog. Click here for file

Additional file 4 **Supplemental Table S4**. Genes common to *P. yoelii, P. berghei *and *P. chabaudi*. Click here for file

Additional file 5 **Supplemental Table S5**. Genes common to both *P. yoelii *and *P. chabaudi*. Click here for file

Additional file 6 **Supplemental Table S6**. Genes common to both *P. yoelii *and *P. berghei*. Click here for file

Additional file 7 **Supplemental Table S7**. *P. yoelii *specific genes. Click here for file

Additional file 8 **Supplemental Table S8**. Primer sequences. Click here for file

Additional file 9 **Supplemental Table S9**. Oligonucleotide probe sequences. Click here for file

Additional file 10 **Supplemental Table S10**. Associative table where predicted rodent parasite gene models are matched with their respective oligonucleotide probes. Click here for file
